# Real-Time Observation of Capsaicin-Induced Intracellular Domain Dynamics of TRPV1 Using the Diffracted X-ray Tracking Method

**DOI:** 10.3390/membranes13080708

**Published:** 2023-07-30

**Authors:** Kazuhiro Mio, Tatsunari Ohkubo, Daisuke Sasaki, Tatsuya Arai, Mayui Sugiura, Shoko Fujimura, Shunsuke Nozawa, Hiroshi Sekiguchi, Masahiro Kuramochi, Yuji C. Sasaki

**Affiliations:** 1 AIST-UTokyo Advanced Operando-Measurement Technology Open Innovation Laboratory (OPERANDO-OIL), National Institute of Advanced Industrial Science and Technology (AIST), 6-2-3 Kashiwanoha, Chiba 277-0882, Japan; 2 Graduate School of Medical Life Science, Yokohama City University, 1-7-29 Suehiro-cho, Tsurumi-ku, Yokohama 230-0045, Japan; 3 Graduate School of Frontier Sciences, The University of Tokyo, 5-1-5 Kashiwanoha, Chiba 277-8561, Japant.arai@edu.k.u-tokyo.ac.jp (T.A.); 4 Photon Factory, Institute of Materials Structure Science, High Energy Accelerator Research Organization, 1-1 Oho, Tsukuba 305-0801, Japan; noz@post.kek.jp; 5 Center for Synchrotron Radiation Research, Japan Synchrotron Radiation Research Institute, 1-1-1 Kouto, Sayo-cho 679-5198, Japan; 6 Graduate School of Science and Engineering, Ibaraki University, Hitachi 316-8511, Japan

**Keywords:** diffracted X-ray tracking technique, TRP channel, conformation dynamics, single molecule analysis, TRPV1

## Abstract

The transient receptor potential vanilloid type 1 (TRPV1) is a multimodal receptor which responds to various stimuli, including capsaicin, protons, and heat. Recent advances in cryo-electron microscopy have revealed the structures of TRPV1. However, due to the large size of TRPV1 and its structural complexity, the detailed process of channel gating has not been well documented. In this study, we applied the diffracted X-ray tracking (DXT) technique to analyze the intracellular domain dynamics of the TRPV1 protein. DXT enables the capture of intramolecular motion through the analysis of trajectories of Laue spots generated from attached gold nanocrystals. Diffraction data were recorded at two different frame rates: 100 μs/frame and 12.5 ms/frame. The data from the 100 μs/frame recording were further divided into two groups based on the moving speed, using the lifetime filtering technique, and they were analyzed separately. Capsaicin increased the slope angle of the MSD curve of the C-terminus in 100 μs/frame recording, which accompanied a shifting of the rotational bias toward the counterclockwise direction, as viewed from the cytoplasmic side. This capsaicin-induced fluctuation was not observed in the 12.5 ms/frame recording, indicating that it is a high-frequency fluctuation. An intrinsiccounterclockwise twisting motion was observed in various speed components at the N-terminus, regardless of the capsaicin administration. Additionally, the competitive inhibitor AMG9810 induced a clockwise twisting motion, which is the opposite direction to capsaicin. These findings contribute to our understanding of the activation mechanisms of the TRPV1 channel.

## 1. Introduction

The transient receptor potential vanilloid 1 (TRPV1) channel is activated by various stimuli, including heat, protons, and vanilloids, and plays a key role in nociception [1,2,3]. TRPV1 is a homotetramer channel, with each subunit containing six transmembrane segments and a pore-forming region. The pore is formed by the assembly of transmembrane segment 5 (S5), a pore loop, and transmembrane segment 6 (S6), surrounded by a voltage sensor-like domain composed of a bundle of four transmembrane helices (S1–S4). Both the N- and C-termini are intracellular, and an ankyrin repeat domain is prominently present at the N-terminus. Cryo-electron microscopy (cryo-EM) has revealed near-atomic resolution structures of TRPV1 [4,5,6], providing a fundamental framework for TRP activation.

Cryo-EM data of various gating stages, along with simulation studies [7,8,9], suggest the occurrence of rotational and twisting motion during gating, but direct detection of these movements has proven technically challenging.

The most controversial issue regarding the cryo-EM structures is that the capsaicin-bound structure was presented as a “closed form”, with the lower gate open but the upper gate closed [5]. Subsequent studies demonstrated the “open form” by applying capsaicin and heat simultaneously [10], but raised a puzzling question of why capsaicin alone fails to induce the open conformation. To address this enigma, we have focused on the investigation of intramolecular motions.

In our previous study, we employed the diffracted X-ray tracking (DXT) technique to measure the dynamics of the extracellular domains of TRPV1. Capsaicin induced rotational bias towards the clockwise (CW) direction, which was speculated to be a channel opening movement, and this bias was sustained in motions with longer lifetime groups [11]. Based on this finding, it was hypothesized that the primary effect of capsaicin involves the opening of the lower gate and an increase in the oscillation frequency of the upper gate, thereby facilitating calcium influx [11].

In the present study, to elucidate the dynamics associate with ligand binding and channel gating, we investigated the motion of the N- and C-terminal domains using a high-speed time-resolved DXT technique with different recording speeds.

## 2. Methods

### 2.1. Plasmid Construct and Protein Purification

Full-length human TRPV1 was subcloned into pcDNA3.1 and tagged with (His)_6_ sequence at the N-terminus and the FLAG sequence (DYKDDDDK) at the C-terminus. The TRPV1 protein was expressed in HEK293F cells and cell surface biotinylation was performed using sulfo-NHS-LC-biotin (Thermo Fisher, MA, USA) according to manufacturer’s instructions. The TRPV1 protein was purified with two-step purification using an anti-FLAG affinity chromatography (Sigma-Aldrich, MO, USA) and a Superdex 200 Increase size exclusion chromatography (Cytiva, MA, USA). The peak fractions were concentrated to 0.1 mg/mL for DXT.

### 2.2. Sample Preparation for DXT Assays

Gold nanocrystals, which were grown on a KCl (100) surface via epitaxial growth under a 10^−4^ Pa vacuum, were dissolved in double-distilled water, and conjugated with anti (His)_6_ monoclonal antibody (clone 9C11, FUJIFILM-Wako, Osaka, Japan) or anti FLAG monoclonal antibody (clone M2, Sigma-Aldrich) under borate buffer, pH 9.0. Buffer component was substituted with recording buffer (PBS (pH 7.4) with 5 mM n-decyl-β-D-maltoside (Sigma-Aldrich)) before applying to TRPV1. For the C-terminal analysis of the 12.5 ms/frame recording, ZnO crystal was used for labeling antibodies.

To immobilize biotinylated TRPV1 proteins to the basement surface, 12.5 μm thick polyimide film (Du Pont-Toray, Tokyo, Japan) was coated with cadmium and chromium (Cd/Cr) by evaporation, then biotin-functionalized self-assembled monolayers (Biotin-SAM) were formed using Biotin-SAM Formation Reagent (Dojindo, Kumamoto, Japan). After binding streptavidin on the Biotin-SAM membrane, twenty microliters of biotinylated TRPV1 (N-terminally (His)_6_-tagged and C-terminally FLAG tagged, ~0.1 mg/mL) were applied and incubated at 4 °C for 6 h. Excess protein was washed out with buffer, and the gold- or ZnO-conjugated antibodies (dispersed in a recording buffer) were applied. After incubation for 20 min at 4 °C, excess antibodies were washed out. Ten microliter of recording buffer containing 10 μM capsaicin with or without 10 μM AMG9810 was applied. The sample chamber was covered with another layer of polyimide film, sandwiched by stainless steel frames and screw clamped. Capsaicin and AMG9810 were purchased from Sigma-Aldrich.

### 2.3. DXT Measurements

DXT measurements were conducted using the SPring-8 BL40XU and the Photon Factory Advanced Ring AR-NW14A beamlines. The X-ray beam size at SPring-8 BL40XU beamline was adjusted to 50 μm in diameter by inserting a pinhole aperture upstream of the sample, and time-resolved diffraction images from the gold nanocrystals were recorded by an X-ray image intensifier (V7739P, Hamamatsu photonics, Hamamatsu, Japan) and a CMOS camera (Phantom V2511, Vision Research, NJ, USA). For each sample, diffractions at 36 positions (6 × 6) within 1 mm^2^ (1 mm × 1 mm) were recorded. The distance between the sample and the detector was set to 45.6 mm. X-rays with an energy bandwidth of 0.1 (15.8 keV in peak energy and photon flux of 10^13^ photons/s) were used for the DXT. Data were recorded with 100 μs/frame intervals and measured for 450 frames per spot. Time-resolved diffraction images at the Photon Factory Advanced Ring AR-NW14A beamline were recorded using a 2D photon-counting detector Pilatus 100 K (Dectris, Baden-Dättwil, Switzerland). The gap of an insertion device U20 was set to 15 mm and the white X-ray with 17.7 keV in peak energy with an energy bandwidth of 0.1 (photon flux of 10^13^ photons/s) was used [12]. The distance between the sample and the detector was set at 95 mm, with beam size of 100 μm × 250 μm. Data were recorded with 12.5 ms/frame intervals and measured for 5000 frames per spot. Recording was performed in triplicate for each sample, with 0.2 mm distance. Temperature at the sample chamber was kept at 25 °C using the Peltier cooling-and-heating stage (Type 10084L, Japan Hightech, Fukuoka, Japan). The concentration of the antibody-gold (or ZnO) conjugate was optimized to obtain approximately 1000 trajectories per recording. X-ray radiation damage was assessed by comparing the MSD curves calculated from the two groups: the first half and the second half.

### 2.4. Image Analysis for DXT

The obtained data were calibrated with the concurrently recorded intensity of incident X-ray (I_0_). Each diffraction spot was tracked by TrackPy (v0.3.2 https://doi.org/10.5281/zenodo.60550) after correcting the background. Trajectories were analyzed using a custom software written within IGOR Pro version 9 (Wavemetrics, Lake Oswego, OR, USA). Diffraction spots of Au (111), ZnO (100), (002), (101) were used for the analysis. The diffractions from the fast-moving proteins have a short time duration between the appearance and disappearance in the observation area, while those of slow-moving proteins have longer duration. For the lifetime filtering technique, trajectories with short lifetime (LT < 2.5 ms) and medium lifetime (2.5 ms ≤ LT < 4.0 ms) were extracted from the 100 μs/frame recording data, and they were analyzed separately.

### 2.5. Mean Squared Displacement Curve

Protein dynamics were analyzed using a mean squared displacement (MSD) algorithm to extract the local behavior of the protein as a function of time. The MSD curves were fitted by the following function: *δ*^2^ (*t*) = *D_α_ t^α^* + 2*β*^2^. *D_α_* is the anomalous diffusion constant, a non-linear relationship to time. *α* represents subdiffusion (1 >> *α* > 0) or superdiffusion (*α* > 1), and *β* is a measurement error.

## 3. Results

**Figure 1:**
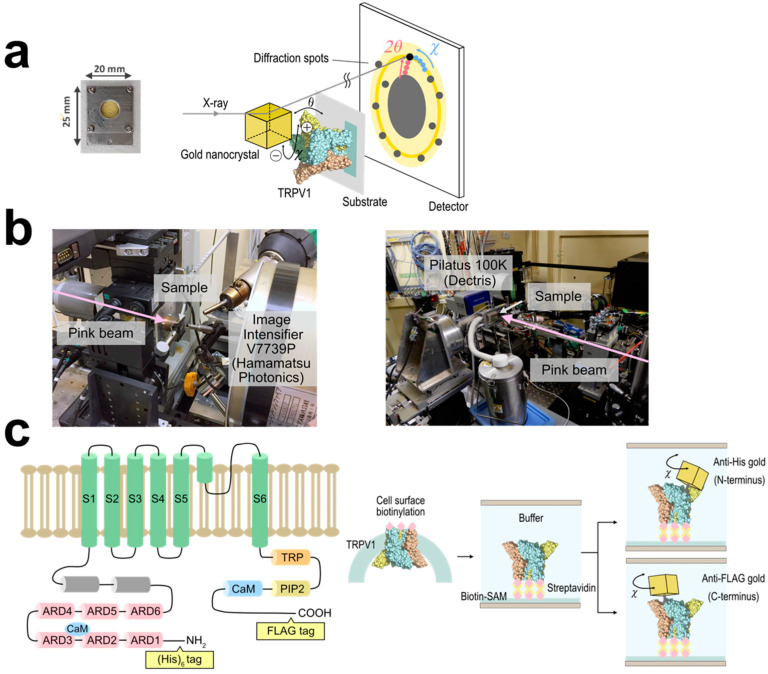
Diffracted X-ray tracking (DXT) measurements. (**a**) Schematic of the DXT measurement. Gold nanocrystals were labeled at specific positions of the target proteins, and their motion was determined by the analysis of the diffraction spots. Pink-beam X-rays elicited trackable diffraction spots from the gold nanocrystals. (**b**) **Left**: Experimental setup for the DXT measurement at the SPring-8 BL40XU beamline. Time-resolved diffraction images from the gold nanocrystals were recorded by an X-ray image intensifier (V7739P, Hamamatsu photonics) and a CMOS camera (Phantom V2511, Vision Research) with 100 μs/frame. **Right**: Photon Factory Advanced Ring AR-NW14A beamline. Data were recorded using a 2D photon-counting detector (Pilatus 100 K, Dectris) with 12.5 ms/frame. (**c**) **Left**: Structure of TRPV1 monomer. The (His)_6_-tag and FLAG-tag were introduced at the N- and C- terminus, respectively. ARD: ankyrin repeat domain, TRP: TRP-box domain, PIP2: PIP2 binding site, CaM: calmodulin binding site. **Right**: Labeling of TRPV1 with nanocrystal-conjugated antibodies and sample preparation. N-terminally (His)_6_-tagged, C-terminally FLAG tagged, and biotinylated TRPV1 was immobilized on a biotin-SAM formed polyimide films.

We analyzed the intracellular domain dynamics of TRPV1 using the DXT technique (Figure 1a). DXT uses X-rays with the wavelength of 0.01 nm to 0.1 nm, enabling positioning accuracy at the picometer level. DXT can also measure dynamics to the order of microseconds to milliseconds, due to the high-brightness X-rays. The motions of the N- and C-termini were investigated separately, using distinct peptide tags and their corresponding antibodies. Gold or ZnO nanocrystals were bound to the antibodies in advance, to obtain diffractions. Diffraction recording was conducted at 100 μs per frame for fast motion and 12.5 ms per frame for slow motion (Figure 1b). The utilization of different frame rates allowed the extraction of distinct motion components. DXT recording with 100 μs/frame was conducted at the SPring-8 BL40XU beamline, and 12.5 ms/frame at the Photon Factory AR-NW14A beamline. The resulting diffraction spots were analyzed in two axes of tilt (θ) (Supplementary Figures S1 and S2 and Supplementary Tables S1 and S2) and twist (χ) angles. For each experimental condition, we analyzed 844–5222 trajectory points, providing a comprehensive assessment of the system.

### 3.1. N-Terminal Dynamics of TRPV1

The N-terminal domain of TRPV1 comprises a typical ankyrin repeat domain (ARD), which is considered to play a major function in thermo-sensation [13,14] as well as modulating channel function by binding with multiple ligands [15,16,17]. The TRPV1 proteins were transiently expressed in HEK293F cells in suspension culture, and their extracellular domain was biotinylated using the surface biotinylation technique (Figure 1c). The TRPV1 protein was purified, and bound to Cd/Cr-coated polyimide films through a biotin-streptavidin reaction, on which biotin-SAM was generated in advance. The motions of the N-terminus were monitored using gold nanocrystal-conjugated antibodies specific to the hexahistidine (His)_6_-tag introduced at the N-terminus.

**Figure 2:**
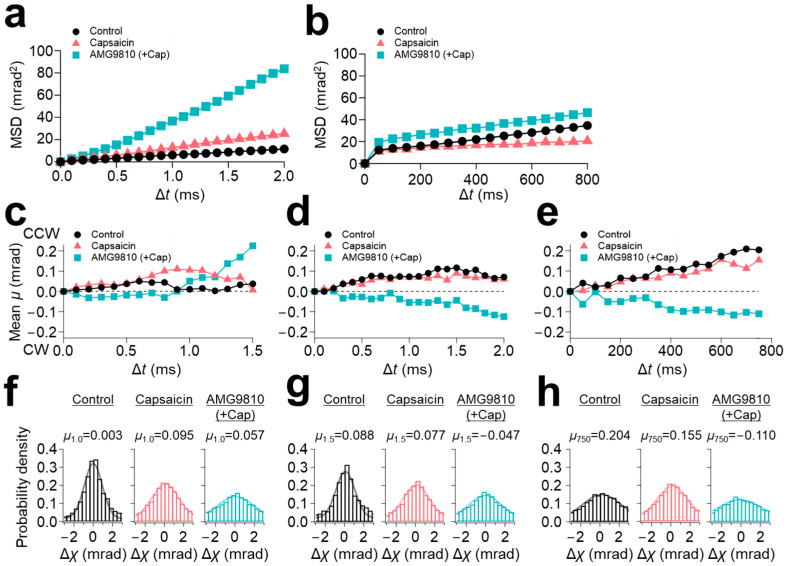
N-terminal domain movement of TRPV1 (χ axis). Mean squared displacement (MSD) curves of TRPV1 N-terminus for the χ axis recorded at (**a**) 100 μs/frame and (**b**) 12.5 ms/frame. (**c**) Rotational angle analysis for the χ axis of short-lifetime group (LT < 2.5 ms, left), (**d**) medium-lifetime group (2.5 ms ≤ LT < 4 ms) at 100 μs/frame recording and (**e**) 12.5 ms/frame recording. (**f**) The distribution of angular displacement of short-lifetime group, (**g**) medium-lifetime group at 100 μs/frame recording, and (**h**) 12.5 ms/frame recording.

Mean square displacement (MSD) curves were calculated for each experiment. MSD curves of 100 μs/frame recording revealed a slight increase in the slope of the curve by capsaicin (5 mrad^2^/ms for control and 10 mrad^2^/ms for capsaicin) (Figure 2a). However, further enhancement of the motion to 40 mrad^2^/ms was observed upon application of AMG9810 (Figure 2a). In contrast, the MSD curves of 12.5 ms/frame recording showed comparable values between control and AMG9810 of 0.04 mrad^2^/ms, but which were suppressed to 0.02 mrad^2^/ms by capsaicin (Figure 2b).

To characterize the motion properties, we performed a directional analysis by segregating the rotational (χ) data into CW and counterclockwise (CCW) directions. The rotational bias was determined from the cytoplasmic side by comparing it to the anchored extracellular domains.

The calculation from the entire dataset did not show an apparent rotational bias, due to the closed sample chamber conditions. Therefore, we applied a lifetime filtering technique [11]. Lifetime was defined as the time between the appearance and disappearance of a diffraction spot in the observation area. Diffraction spots can be recorded within the energy range of synchrotron radiation; the fast-moving proteins have a short time duration while slow-moving proteins have longer duration. Therefore, the duration of diffraction spots (lifetime) represents the overall movement of the target protein, providing ligand-induced internal motility of TRPV1. Trajectories with lifetimes below 2.5 ms (LT < 2.5 ms) represent very fast movements, and trajectories with lifetimes between 2.5 ms and 4.0 ms (2.5 ms ≤ LT < 4.0 ms) represent medium-speed movements. They were extracted from the original dataset and analyzed separately.

We successfully extracted capsaicin-induced rotational bias in the domains. The short-lifetime group (LT < 2.5 ms) exhibited a slight CCW bias for all three conditions (Figure 2c). The medium-lifetime group (2.5 ms ≤ LT < 4.0 ms) demonstrated a sustained CCW bias for the control and capsaicin, but represented the opposite direction of CW bias for AMG9810 (Figure 2d). The rotational bias obtained from the 12.5 ms/frame recording was similar to those of the medium-speed movement group (2.5 ms ≤ LT < 4.0 ms): CCW rotational bias for both control and capsaicin, and CW rotational bias for AMG9810 (Figure 2e).

The rotation bias analysis demonstrated that the N-terminal movement of control and capsaicin comprises various moving speed components having CCW rotational biases (Figure 2c–e). It should be noted that these movements were recorded within the limited recording frame rate windows, and there may exist other motion components outside the windows; probably, much slower untwisting movements may be occurring as well.

The distribution of movement along the χ axis was displayed by histograms and fitted by Gaussian curves. Peak positions of all three curves of the fast-movement group (LT < 2.5 ms) were slightly positive (0.003 for control, 0.095 for capsaicin, and 0.057 for AMG9810) (Figure 2f). The distributions were broadened by capsaicin and further broadened by AMG9810 (full width at half maximum (FWHM) were: 2.191 for control, 3.316 for capsaicin, and 4.270 for AMG9810; Table 1), reflecting an increase in motion for capsaicin and AMG9810, as observed in the Figure 2a. The histograms were similar for the medium-speed movement group (2.5 ms ≤ LT < 4.0 ms), but the peak position of AMG9810 was shifted to the negative value (0.088 for control, 0.077 for capsaicin, and −0.047 for AMG9810).

As for the slow-speed motion (12.5 ms/frame recording), the peak position was not significantly influenced by capsaicin (0.204 for control and 0.155 for capsaicin), but the FWHM was decreased (4.653 for control and 3.740 for capsaicin), suggesting that capsaicin reduced the movement at the N-terminus, as observed in the Figure 2b. The peak position of the AMG9810 was shifted to −0.110.

In conclusion, capsaicin did not induce significant motion change to the N-terminal domain, and, regardless of the frame rates, both the control and capsaicin intrinsically had a CCW rotational bias. On the other hand, AMG9810 exhibited a clear increase in the motion with opposite directional movement compared to the control and capsaicin. This trend was typically observed in the motion of the medium- and slow-speed groups. It is speculated that the competitive inhibitor AMG9810 inhibits the endogenous rotational movement in TRPV1, resulting in the extraction of only the CW directional movement.

### 3.2. C-Terminal Dynamics of TRPV1

**Figure 3:**
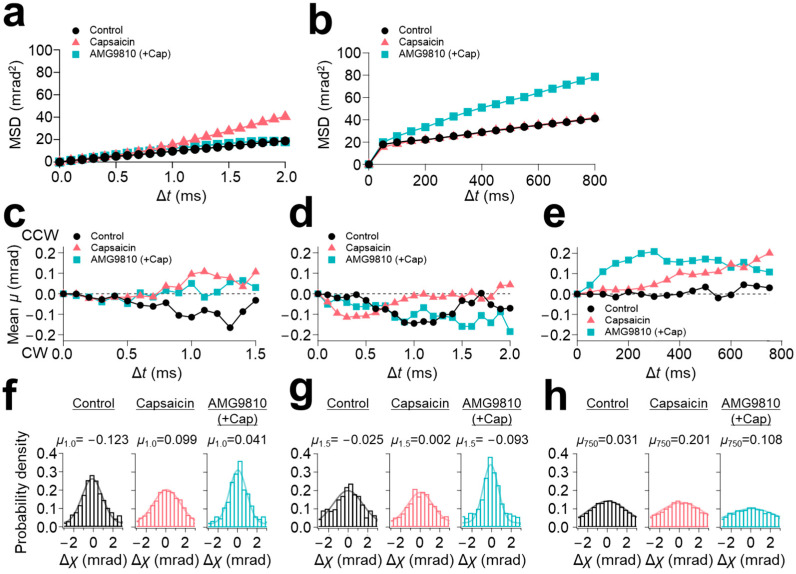
C-terminal domain movement of TRPV1 (χ axis). Mean squared displacement (MSD) curves of TRPV1 C-terminus for the χ axis recorded at (**a**) 100 μs/frame and (**b**) 12.5 ms/frame. (**c**) Rotational angle analysis for the χ axis of short-lifetime group (LT < 2.5 ms, left), (**d**) medium-lifetime group (2.5 ≤ LT < 4 ms) at 100 μs/frame recording and (**e**) 12.5 ms/frame recording. (**f**) The distribution of angular displacement of short-lifetime group, (**g**) medium-lifetime group at 100 μs/frame recording, and (**h**) 12.5 ms/frame recording.

The C-terminal domain of TRPV1 locates much closer to the gate in the sequence. The S6 segment undergoes conformational changes upon capsaicin binding [5]. The S6 segment leads to the TRP-box domain, which forms a long helix parallel to the membrane plane and to the cytoplasmic C-terminus. The C-terminal domain contains calmodulin and phosphatidylinositol binding sites, which are considered to modulate channel function [18,19]. The C-terminal domain is mostly intrinsically disordered. The MSD curves from the 100 μs/frame recording demonstrated that capsaicin increased the slope angle from 10 mrad^2^/ms (control) to 20 mrad^2^/ms at the C-terminus (Figure 3a). However, in contrast to the N-terminus, AMG9810 did not enhance the slope angle. As for the 12.5 ms/frame recording, capsaicin did not change the slope angle of the MSD curve (Figure 3b); instead, AMG9810 increased the slope angle, as observed in the N-terminus.

These dynamics were further analyzed using the lifetime filtering technique and the rotational bias analysis. Capsaicin induced CCW rotational bias for the fast-movement group (LT < 2.5 ms) (Figure 3c), consequently shifting the peak position of capsaicin in the histograms from −0.123 (control) to 0.099 (Figure 3f). In contrast to the broadening of the histograms at the N-terminus, the histograms of AMG9810 in both the short-lifetime group (LT < 2.5 ms) and medium-lifetime group (2.5 ms ≤ LT < 4.0 ms) were narrowed, representing a decrease in motion dynamics (Figure 3f,g and Table 2).

As for the slow-speed motion (12.5 ms/frame recording), all three conditions showed CCW rotational biases (Figure 3e). Gaussian fitting for these conditions did not show a significant change in distributions (Figure 3h). The change in the peak position was also limited (0.031 for control, 0.201 for capsaicin, and 0.108 for AMG9810).

In conclusion, the motion of the C-terminal domain was highly influenced by the ligand. In the 100 μs/frame recording, capsaicin induced an increase in movement, accompanied by a significant shift in the rotational bias towards CCW direction. This movement was considered a very fast oscillatory movement, as it was not observed in other timescale data. Considering the timescale, this movement can be related to the gating dynamics. On the other hand, AMG9810 suppressed the capsaicin-induced motion at the C-terminus in the 100 μs/frame recording. This is in sharp contrast to the AMG9810 at the N-terminus, where it increased movement, accompanied by a clear rotational bias. Histogram analysis also demonstrated that AMG9810 effectively suppressed the C-terminal motion.

### 3.3. Subtraction Analysis for Capsaicin-Induced Intracellular Domain Dynamics of TRPV1

To elucidate the capsaicin-induced rotational (χ) domain movement more clearly, the histogram subtractions between them, with and without capsaicin, were performed and analyzed.

**Figure 4:**
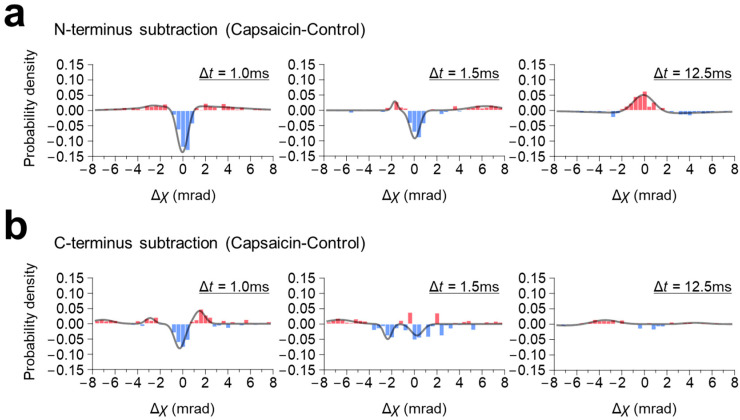
Subtraction maps of the distribution of angular displacement. Maps were generated by subtracting the population distribution histograms of control (without capsaicin) from those of capsaicin. (**a**) Motion difference at the N-terminus, and (**b**) the C-terminus. From left to right: subtraction maps of the short-lifetime group (LT < 2.5 ms), medium-lifetime group (2.5 ms ≤ LT < 4 ms) at 100 μs/frame recording, and 12.5 ms/frame recording.

At the N-terminus, capsaicin did not induce significant change in rotational bias, but broadened the population distribution for both the fast- (LT < 2.5 ms) and the medium-moving groups (2.5 ms ≤ LT < 4.0 ms) in the 100 μs/frame recording, and narrowed it in the 12.5 ms/frame recording (Figure 2). Consequently, the subtraction graphs of both the fast- (LT < 2.5 ms) and medium-moving groups (2.5 ms ≤ LT < 4.0 ms) showed a single negative peak around zero mrad, with small positive peaks on both sides, indicating that capsaicin increased the MSD curve angle without changing the rotational bias (Figure 4a, Supplementary Table S3). In contrast to the 100 μs/frame, the subtraction histogram of the 12.5 ms/frame recording showed a single positive peak around the zero mrad, suggesting that capsaicin narrowed the population distribution without changing the rotational bias.

In contrast, capsaicin shifted the rotational bias at the C-terminus in the 100 μs/frame recording (Figure 3c). This shift was typically observed in the fast-moving group (LT < 2.5 ms), where the peak position of capsaicin was shifted CCW from −0.123 mrad (control) to 0.099 mrad (Figure 3f), resulting in the two-peak curve with one negative peak at the −0.28 mrad and one positive peak at the 1.48 mrad (Figure 4b), Supplementary Table S4). The capsaicin-induced rotational bias was not obvious in the subtraction maps of the medium-moving group (2.5 ms ≤ LT < 4.0 ms) and the 12.5 ms/frame recording.

## 4. Discussion

**Figure 5:**
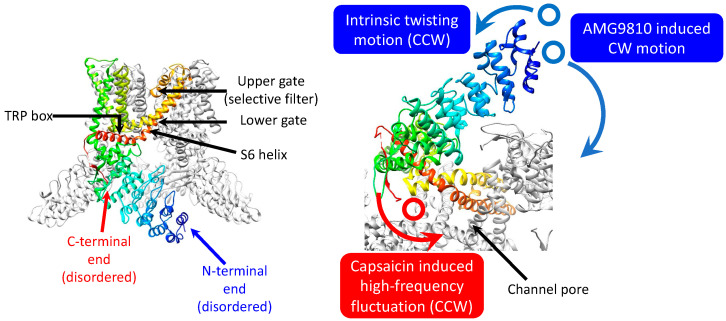
Motion dynamics of TRPV1 cytoplasmic domain activation by capsaicin. **Left**: A monomer subunit from the TRPV1 tetramer is depicted in rainbow color scheme from the N-terminus (blue) to the C-terminus (red). Data are displayed by the UCSF Chimera version 1.15 (PDB: 3J5P). **Right**: DXT demonstrates motion dynamics of the N- and C-terminal domains viewed from the cytoplasmic side.

In this study, we applied the DXT technique to understand the intracellular domain dynamics of the TRPV1 channel. DXT is a very powerful method for clarifying membrane protein dynamics [11,20,21,22], analyzing the dynamics of functional proteins like chaperonin [23], and analyzing the oligomerization mechanisms of amyloid β (Aβ) [24], the Alzheimer’s disease-related protein; it is also able to do this with a high spatiotemporal resolution, even for the intramolecular dynamics in living cells [25,26]. Capsaicin induced high-frequency fluctuational movement at the C-terminus, while CCW twisting motion on a range of various timescales was observed at the N-terminus, regardless of the addition of capsaicin (Figure 5). The rotational bias observed in the control may reduce the energy barrier in gating, which may be beneficial for capsaicin to pull the trigger for gating, with minimum conformational changes.

The N-terminal domain of a TRPV1 monomer comprises six ankyrin repeats; each ankyrin repeat consists of two antiparallel α helices (inner and outer helix), separated by hairpin loops. The ARD of TRPV1 comprises calmodulin and ATP binding sites, and the channel function is modulated by binding with Ca^2+^, ATP and other multiple ligands [15,16,17]. Recent studies also suggested that the ARD plays an important role in thermal sensation [13,14]. Point mutations in TRPV1 ARD induce drastic changes in the temperature threshold in tailed amphibians [14].

In our experiment, capsaicin did not show significant influence on the N-terminal domain motion, and, independently, an intrinsic CCW rotational bias was observed for the control and capsaicin. Because recent studies suggested that the N-terminal ARD has a major function in thermal sensation, further studies are expected to apply DXT to analyze the thermo-induced TRPV1 gating mechanisms. We also observed that AMG9810 exhibited a clear increase in the MSD curve, but its rotational bias was in the opposite direction compared to the control and capsaicin. We have confirmed in advance that the AMG9810 inhibits capsaicin-induced calcium entry, using the cell-based calcium influx assay. It is speculated that the competitive inhibitor AMG9810 inhibits the endogenous rotational movement in TRPV1, resulting in the extraction of only the opposing directional movement.

The C-terminal part of a TRPV1 monomer comprises the TRP-box domain, which is conserved in the TRPC, TRPV, and TRPM subfamilies [27]. The TRP-box is a long helix, parallel to the membrane plane [4]. The gating of the TRPV1 channel is regulated by the PIP_2_ and calmodulin bindings and by phosphorylation at the TRP-box and other part of the C-terminus [18,19]. However, most of the cytoplasmic C-terminus is intrinsically disordered, and its structure is not well understood.

In our experiment, the C-terminal domain motion was highly influenced by ligands. In the 100 μs/frame recording, capsaicin increased the movement at the C-terminus, accompanied by a significant shift in the rotational bias towards the CCW direction. This movement was considered a very fast oscillatory movement, because it was not obvious in other timescale data. Conformational dynamics of ion channels occur on nanosecond-to-millisecond timescales. Considering the timescale, this movement can be associated with gating-related molecular dynamics. Further studies using functionally modified mutant proteins or introducing protein decoration, including phosphorylation, calmodulin and PIP_2_ binding, may be helpful in elucidating the detailed TRPV1 gating mechanisms.

Our previous study using DXT to measure the extracellular domain dynamics of TRPV1 demonstrated that capsaicin induced a CW rotational bias, which was suggested as a gate-opening movement [11]. From these results, capsaicin may primarily open the lower gate by the twisting movement of the transmembrane helices and increase the oscillation frequency of the upper gate as observed in the C-terminal end, facilitating calcium influx. Further study is needed to fully understand TRPV1’s gating mechanism and interaction dynamisms by capsaicin and other ligands.

In our analysis, measurements were conducted at different frame rates. We also introduced the lifetime filtering technique, which can successfully classify the motions by their moving speed. Different frame rates captured motion components operating at significantly different speeds, including rapid conformational changes on microsecond-to-millisecond time scales. Patch clamp experiments suggested that a half-activation time of TRPV1 by capsaicin is more than hundred milliseconds, significantly slower than the activation by heat at 51 °C (*t*1/2 = ~6 ms) [28]. Our DXT experiments were performed at frame rates from 100 μs to 12.5 ms, covering the timescale for gate opening expected from the patch clamp experiments. Patch clamp measures ion permeation resulting from gate opening, whereas DXT detects all motions related to gating. In combination with the patch clamp technique, DXT may be a powerful tool for unraveling the mechanisms behind, such as pore dilation (agonist stimulation-induced ionic selectivity changes) [29]. By introduced this technique, we could obtain the capsaicin-induced rotational bias at the intracellular domains, which was not evident in the statistical analysis using the unfiltered data source.

## Figures and Tables

**Table 1:** Gaussian fitting parameters for the N-terminus (χ).

	Control	Capsaicin	AMG9810 (+Cap)
LT < 2.5 ms (100 μs/frame recording)			
-Location (mrad)	0.003 ± 0.032	0.095 ± 0.034	0.057 ± 0.054
-FWHM (mrad)	2.191 ± 0.081	3.316 ± 0.090	4.270 ± 0.156
-Peak area	0.713 ± 0.026	0.674 ± 0.019	0.546 ± 0.022
2.5 ms ≦ LT < 4.0 ms (100 μs/frame recording)			
-Location (mrad)	0.088 ± 0.044	0.077 ± 0.038	−0.047 ± 0.058
-FWHM (mrad)	2.317 ± 0.110	3.135 ± 0.101	3.837 ± 0.160
-Peak area	0.637 ± 0.030	0.618 ± 0.020	0.508 ± 0.023
12.5 ms/frame recording			
-Location (mrad)	0.204 ± 0.037	0.155 ± 0.037	−0.110 ± 0.055
-FWHM (mrad)	4.653 ± 0.112	3.740 ± 0.101	5.011 ± 0.173
-Peak area	0.671 ± 0.019	0.723 ± 0.021	0.554 ± 0.023

**Table 2:** Gaussian fitting parameters for the C-terminus (χ).

	Control	Capsaicin	AMG9810 (+Cap)
LT < 2.5 ms (100 μs/frame recording)			
-Location (mrad)	−0.123 ± 0.037	0.099 ± 0.028	0.041 ± 0.040
-FWHM (mrad)	2.456 ± 0.095	3.237 ± 0.075	2.093 ± 0.101
-Peak area	0.639 ± 0.024	0.647 ± 0.016	0.656 ± 0.031
2.5 ms ≦ LT < 4.0 ms (100 μs/frame recording)			
-Location (mrad)	−0.025 ± 0.081	0.002 ± 0.053	−0.093 ± 0.042
-FWHM (mrad)	3.537 ± 0.221	3.007 ± 0.139	1.827 ± 0.104
-Peak area	0.690 ± 0.046	0.536 ± 0.025	0.619 ± 0.033
12.5 ms/frame recording			
-Location (mrad)	0.031 ± 0.028	0.201 ± 0.048	0.108 ± 0.058
-FWHM (mrad)	4.492 ± 0.082	5.109 ± 0.152	5.496 ± 0.197
-Peak area	0.598 ± 0.012	0.646 ± 0.023	0.491 ± 0.022

## Data Availability

Not applicable.

## Supplementary Materials

The following supporting information can be downloaded at: https://www.mdpi.com/article/10.3390/membranes13080708/s1, Figure S1: N-terminal domain movement of TRPV1 (θ axis); Figure S2: C-terminal domain movement of TRPV1 (θ axis); Table S1: Gaussian fitting parameters for the N-terminus (θ); Table S2: Gaussian fitting parameters for the C-terminus (θ); Table S3: Fitting parameters for the subtraction analysis (N-terminus, χ); Table S4: Fitting parameters for the subtraction analysis (C-terminus, χ).
