# Supplementary material for: Real-Time Observation of Capsaicin-Induced Intracellular Domain Dynamics of TRPV1 Using the Diffracted X-ray Tracking Method
Source: Membranes (Basel). 2023 Jul 30;13(8):708. doi: 10.3390/membranes13080708 (PMC10456751; doi:10.3390/membranes13080708)
Supplement: Supplementary file 1 [file membranes-13-00708-s001.zip › membranes-2522323-supplementary.pdf]

Supplementary information

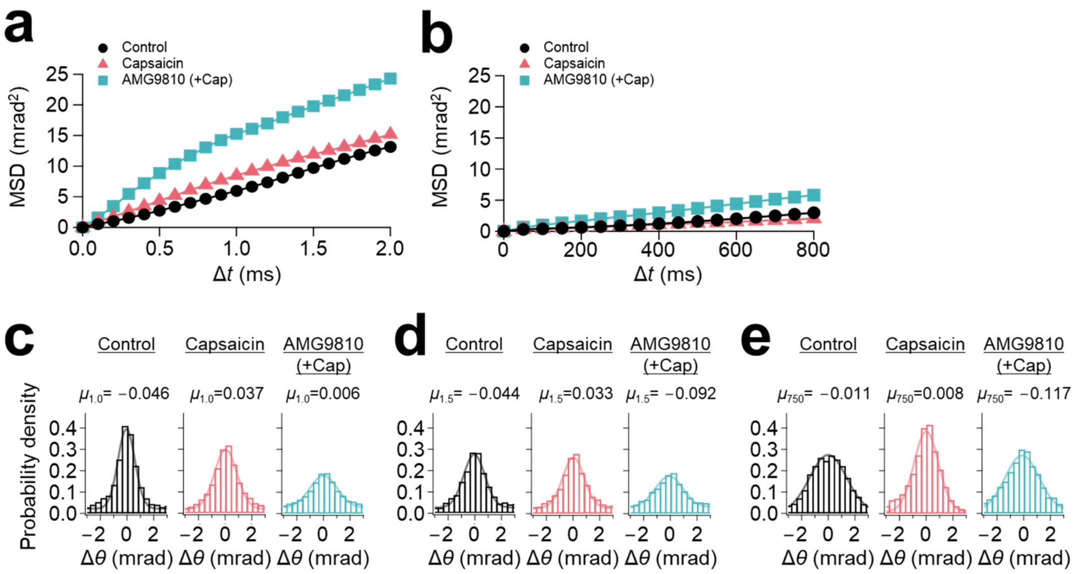

Supplementary Figure S1. N-terminal domain movement of TRPV1 (θ axis)

(a) Mean square displacement (MSD) curves of TRPV1 N-terminus for the θ axis recorded at 100 μs/frame and (b) 12.5 ms/frame. (c) The distribution of angular displacement of short lifetime group (LT < 2.5 ms, left), (d) medium lifetime group (2.5 ms ≤ LT < 4 ms) at 100 μs/frame recording and (e) 12.5 ms/frame recording.

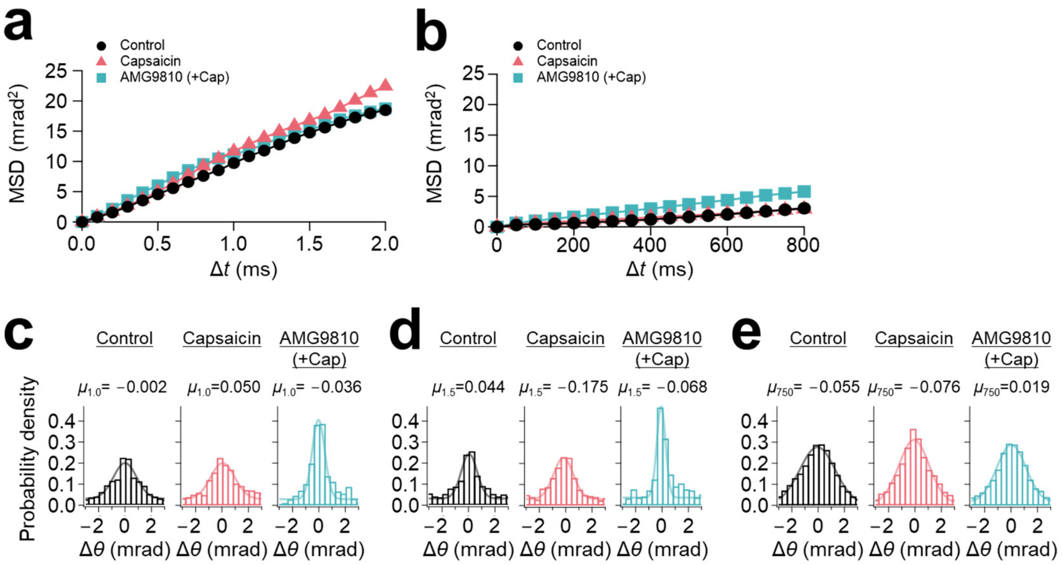

Supplementary Figure S2. C-terminal domain movement of TRPV1 (θ axis)

(a) Mean square displacement (MSD) curves of TRPV1 C-terminus for the θ axis recorded at 100 μ s/frame and (b) 12.5 ms/frame. (c) The distribution of angular displacement of short lifetime group (LT < 2.5 ms, left), (d) medium lifetime group (2.5 ms ≤ LT < 4 ms) at 100 μ s/frame recording and (e) 12.5 ms/frame recording.

Supplementary Table S1. Gaussian fitting parameters for the N-terminus (θ)

	Control	Capsaicin	AMG9810 (+Cap)
LT < 2.5 ms (100 μs/frame recording)			
-Location (mrad)	-0.046 ± 0.022	0.037 ± 0.025	0.006 ± 0.040
-FWHM (mrad)	1.504 ± 0.055	1.973 ± 0.063	2.424 ± 0.103
-Peak area	0.598 ± 0.020	0.587 ± 0.018	0.388 ± 0.016
2.5 ms ≦ LT < 4.0 ms (100 μs/frame recording)			
-Location (mrad)	-0.044 ± 0.026	0.033 ± 0.028	-0.092 ± 0.044
-FWHM (mrad)	1.873 ± 0.065	1.901 ± 0.071	2.404 ± 0.112
-Peak area	0.498 ± 0.016	0.478 ± 0.017	0.372 ± 0.017
12.5 ms/frame recording			
-Location (mrad)	-0.011 ± 0.021	0.008 ± 0.031	-0.117 ± 0.038
-FWHM (mrad)	3.266 ± 0.057	2.160 ± 0.077	3.004 ± 0.100
-Peak area	0.941 ± 0.017	0.867 ± 0.030	0.837 ± 0.028

Supplementary Table S2. Gaussian fitting parameters for the C-terminus (θ)

	Control	Capsaicin	AMG9810 (+Cap)
LT < 2.5 ms (100 μs/frame recording)			
-Location (mrad)	-0.002 ± 0.052	0.050 ± 0.054	-0.036 ± 0.026
-FWHM (mrad)	2.052 ± 0.131	2.156 ± 0.137	1.126 ± 0.064
-Peak area	0.370 ± 0.023	0.393 ± 0.024	0.451 ± 0.023
2.5 ms ≦ LT < 4.0 ms (100 μs/frame recording)			
-Location (mrad)	0.044 ± 0.049	-0.175 ± 0.032	-0.068 ± 0.025
-FWHM (mrad)	1.415 ± 0.120	1.756 ± 0.078	0.782 ± 0.062
-Peak area	0.319 ± 0.025	0.366 ± 0.015	0.361 ± 0.025
12.5 ms/frame recording			
-Location (mrad)	-0.055 ± 0.031	-0.076 ± 0.038	0.019 ± 0.028
-FWHM (mrad)	3.153 ± 0.081	2.680 ± 0.099	2.588 ± 0.072
-Peak area	0.910 ± 0.024	0.864 ± 0.032	0.751 ± 0.021

Supplementary Table 3. Fitting parameters for the subtraction analysis (N-terminus , χ)

	Peak1	Peak2	Peak3	Peak4
LT < 2.5 ms (100 μs/frame recording)				
-Location (mrad)	-2.685 ± 0.530	-0.037 ± 0.020	0.671 ± 0.864	
-FWHM (mrad)	1.319 ± 1.475	1.137 ± 0.057	9.391 ± 1.832	
-Peak area	0.009 ± 0.011	-0.183 ± 0.011	0.145 ± 0.027	
2.5 ms ≦ LT < 4.0 ms (100 μs/frame recording)				
-Location (mrad)	-1.715 ± 0.090	0.045 ± 0.032	6.324 ± 0.467	
-FWHM (mrad)	0.562 ± 0.186	1.030 ± 0.075	3.287 ± 1.230	
-Peak area	0.018 ± 0.005	-0.101 ± 0.006	0.048 ± 0.014	
12.5 ms/frame recording				
-Location (mrad)	-0.089 ± 0.079	0.960 ± 1.534		
-FWHM (mrad)	2.173 ± 0.241	12.484 ± 5.645		
-Peak area	0.139 ± 0.020	-0.126 ± 0.032		

Supplementary Table S4. Fitting parameters for the subtraction analysis (C-terminus , χ)

	Peak1	Peak2	Peak3	Peak4
LT < 2.5 ms (100 μs/frame recording)				
-Location (mrad)	-7.093 ± 0.595	-2.902 ± 0.193	-0.280 ± 0.052	1.482 ± 0.101
-FWHM (mrad)	2.127 ± 1.532	0.943 ± 0.454	1.083 ± 0.131	1.115 ± 0.255
-Peak area	0.030 ± 0.018	0.019 ± 0.008	-0.093 ± 0.009	0.050 ± 0.010
2.5 ms ≦ LT < 4.0 ms (100 μs/frame recording)				
-Location (mrad)	-6.616 ± 1.190	-2.364 ± 0.147	0.225 ± 0.239	
-FWHM (mrad)	2.737 ± 3.145	0.822 ± 0.362	1.245 ± 0.564	
-Peak area	0.040 ± 0.037	-0.043 ± 0.016	-0.050 ± 0.020	
12.5 ms/frame recording				
-Location (mrad)	-6.971 ± 1.940	-3.469 ± 0.698	4.528 ± 0.926	
-FWHM (mrad)	2.942 ± 8.866	2.619 ± 1.377	0.011 ± 0.009	
-Peak area	-0.013 ± 0.036	0.037 ± 0.022	2.424 ± 2.180	
